# Three-dimensional modeling of chromatin structure from interaction frequency data using Markov chain Monte Carlo sampling

**DOI:** 10.1186/1471-2105-12-414

**Published:** 2011-10-25

**Authors:** Mathieu Rousseau, James Fraser, Maria A Ferraiuolo, Josée Dostie, Mathieu Blanchette

**Affiliations:** 1 McGill Centre for Bioinformatics, Bellini Building, Life Sciences Complex, 3649 Promenade Sir William Osler, Montréal, Québec, H3G 0B1, Canada; 2 Department of Biochemistry, and Goodman Cancer Research Centre, McGill University, 3655 Promenade Sir-William-Osler, Montréal, Québec, H3G 1Y6, Canada

## Abstract

**Background:**

Long-range interactions between regulatory DNA elements such as enhancers, insulators and promoters play an important role in regulating transcription. As chromatin contacts have been found throughout the human genome and in different cell types, spatial transcriptional control is now viewed as a general mechanism of gene expression regulation. Chromosome Conformation Capture Carbon Copy (5C) and its variant Hi-C are techniques used to measure the interaction frequency (IF) between specific regions of the genome. Our goal is to use the IF data generated by these experiments to computationally model and analyze three-dimensional chromatin organization.

**Results:**

We formulate a probabilistic model linking 5C/Hi-C data to physical distances and describe a Markov chain Monte Carlo (MCMC) approach called *MCMC5C *to generate a representative sample from the posterior distribution over structures from IF data. Structures produced from parallel MCMC runs on the same dataset demonstrate that our MCMC method mixes quickly and is able to sample from the posterior distribution of structures and find subclasses of structures. Structural properties (base looping, condensation, and local density) were defined and their distribution measured across the ensembles of structures generated. We applied these methods to a biological model of human myelomonocyte cellular differentiation and identified distinct chromatin conformation signatures (CCSs) corresponding to each of the cellular states. We also demonstrate the ability of our method to run on Hi-C data and produce a model of human chromosome 14 at 1Mb resolution that is consistent with previously observed structural properties as measured by 3D-FISH.

**Conclusions:**

We believe that tools like *MCMC5C *are essential for the reliable analysis of data from the 3C-derived techniques such as 5C and Hi-C. By integrating complex, high-dimensional and noisy datasets into an easy to interpret ensemble of three-dimensional conformations, *MCMC5C *allows researchers to reliably interpret the result of their assay and contrast conformations under different conditions.

**Availability:**

http://Dostielab.biochem.mcgill.ca

## Background

In the nucleus, genomic DNA exists in the form of chromatin, which is tightly packaged and organized into higher-level structures required for proper genome function [1,2]. Chromatin conformation is highly dynamic and modified by several biological processes such as DNA replication, repair and transcription. The three-dimensional chromatin organization itself was recently found to play an important role in transcription regulation [3-5] and can be used to define chromatin signatures [6-9]. For example, it was shown that elements that lie far apart in the one-dimensional genomic sequence or on different chromosomes could functionally interact through physical contacts [10-12]. One such example is the 100-kb imprinted *Igf2/H19 *locus on human chromosome 11 where there exists an imprinting control region (ICR) located between the *Igf2 *gene and its enhancer sequence. On the maternal allele, CTCF (a known insulator protein) is able to bind the unmethylated ICR and subsequently forms multiple long-range looping contacts along the locus that block gene-enhancer interaction. However, the paternal ICR is methylated and cannot be bound by CTCF, thus allowing the *Igf2 *gene and its enhancer sequence to interact through a long-range loop, thereby regulating expression to only the paternal allele [13-16]. Such long-range interactions have been found throughout metazoan genomes where thus far many of them appear to correlate well with the transcriptional state of target genes [6,17-20].

Although we still do not know how many types of contacts exist or how the majority of them are regulated, it is now clear that spatial transcriptional control is an important mechanism of gene regulation. Thus, mapping of physical contacts within (*cis*) and between (*trans*) chromosomes will be essential to fully understand gene regulation.

Several techniques are now available to examine chromatin structure at high-resolution, such as DamID [21], and more recent approaches including Chromosome Conformation Capture (3C) [22], Circular Chromosome Conformation Capture (4C) [23,24], Chromosome Conformation Capture Carbon Copy (5C) [25], Chromatin interaction analysis with paired-end tag sequencing (ChIA-PET) [26], the technology developed by Duan *et al*. [27], and Hi-C [18]. These techniques combine various high-throughput approaches, such as microarrays and next-generation sequencing, and produce large datasets. In the case of 5C and Hi-C, the measurements obtained consist of pairwise interaction frequency values that are proportional to the proximity of the chromatin fragments in the nuclear space *in vivo*. These data broadly define the three-dimensional conformation of chromatin. It is important to note that these assays are not performed on a single cell, but rather a population of cells, and these data thereby represent population-average measurements of the degree of interaction between chromatin fragments that require tailored bioinformatics tools for interpretation. In this paper, we propose a computational approach to robustly infer ensembles of chromatin conformations that are supported by a given 5C or Hi-C dataset. These three-dimensional models of chromatin conformation can be analyzed to determine robust structural properties.

Recently, several approaches have been proposed to model chromatin 3D conformation from interaction frequency (IF) data. In previous work [19], we developed a program called *5C3D *that first translates IF values into physical distance estimates and then uses a gradient descent approach to find the 3D conformation with the best fit to the observed data based on a simple misfit objective function. Bau *et al*. [17,18] proposed 3D models of the *α*-globin locus based on 5C data. They formulate an optimization problem where pairwise interactions are modeled with springs whose equilibrium length depends on the observed IF values, subject to certain constraints based on the structure of the 30-nm fiber. They then use the Integrative Modeling Platform (IMP; http://salilab.org/imp/) to produce a set of possible conformations that satisfy the constraints while maximizing the fit to the IF data. Duan *et al*. [27] proceed similarly to obtain a model of the budding yeast chromatin conformation based on data obtained using a modification of the 4C technology coupled with high-throughput sequencing. They first convert observed interaction frequencies to Euclidean distances and then seek the chromatin conformation that minimizes the same measure of misfit as *5C3D*, with the addition of a set of clash avoidance constraints, and a few biologically-motivated constraints based on prior knowledge about the yeast genome organization. The constrained optimization problem is solved using an optimization package to produce the best fitting structure. A very similar approach is used by Tanizawa *et al*. [29] to model the genome of fission yeast. Of all these approaches, *5C3D *is the only one we are aware of that comes with stand-alone software.

Although these approaches differ slightly in the manner in which IF data is translated into distance constraints, the set of additional constraints included in the model, and the way the resulting system of equations is solved, they all have the merit of turning a set of noisy IF measurements into a more interpretable read out. By integrating *O*(*n*^2^) noisy IF measurements into *O*(*n*) predictions about the 3D location of each fragment, they also potentially produce an output that is more reliable than any of the individual IF measurements it is based on. However, these approaches suffer from two significant drawbacks. First, the objective function (always some form of sum-of-squared differences between predicted and IF-derived distance) is debatable, as, among other things, it assumes that each IF measurement is equally reliable. Second, the structures obtained come with no guarantee of representativity or reliable measure of uncertainty. Acknowledging this limitation, Baú *et al*. [17,28] proposed a heuristic approach to generate sets of candidate structures. However, because none of these approaches are based on a probabilistic model integrating an IF noise model, the set of sampled structures may not be representative of the true (probabilistically weighted) set of possible structures. Even though the approach used by Baú *et al*. produces an ensemble of solutions, the absence of an underlying probabilistic model prevents the calculation of confidence intervals on specific structural properties (e.g. the distance between two sites along the genome) and do not identify statistically significant conformational features.

In this paper, we introduce *MCMC5C*, a computational probabilistic modeling approach for inferring chromatin three-dimensional structure from 5C or Hi-C experiments. Our approach is based on a formal probabilistic model of interaction frequencies and their link with physical distance and uses a Markov chain Monte Carlo sampling procedure to produce an ensemble of candidate conformations for a given 5C dataset. Unlike gradient descent approaches, *MCMC5C *allows (at least in theory) a proper sampling of the structure state space. This set of structures can be used to obtain posterior distributions over specific structural properties, contrast structural properties of chromatin under different conditions, or determine the existence of multiple model subclasses that fit the experimental data.

Markov chain Monte Carlo approaches have been widely applied to numerous computational and biological problems, such as the prediction of RNA structure [30,31] or protein structure [32,33], phylogenetic inference [34,35], and sequence alignment [36,37]. Our particular application shares some resemblance with the problem of inferring protein structure from nuclear magnetic resonance (NMR) data, which measures distances between hydrogen atoms in a molecule [38,39]. Although existing software for NMR-based protein structure prediction are not applicable to our problem because they are tightly based on specifics of NMR data and amino acid structures, MCMC approaches are commonly used to produce robust ensembles of candidate structures based on noisy distance data.

The rest of this paper is structured as follows. After a brief introduction to the 5C and Hi-C technologies, we introduce a probabilistic model of the link between 5C or Hi-C data and 3D chromatin conformation. We then describe a MCMC-based algorithm that quickly produces an ensemble of structures, and then show how key features of the chromatin structure can be robustly estimated. Our approach is used for the analysis of three 5C datasets generated for the region of human chromosome 7 containing the HoxA gene cluster in both undifferentiated myelomonocytes and differentiated macrophages, revealing key changes in chromatin conformation. We also show that the *MCMC5C *program can be applied to Hi-C data by generating a three-dimensional model of human chromosome 14 at a 1 Mb resolution from previously published data [18].

### Summary of Chromosome Conformation Capture Carbon Copy (5C) and Hi-C technologies

**Figure 1:**
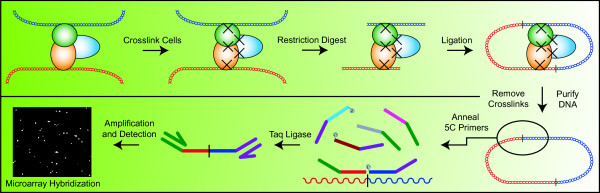
**5C Technology**. Schematic description of Chromosome Conformation Capture Carbon Copy (5C) technology. Illustrated are two strands of DNA in vivo (blue and red double helix), which are bound together by a protein complex (trio of colored spheres). Cells are first crosslinked, which covalently links the protein complex and DNA together. Next, a restriction enzyme is used to cut the DNA at very specific locations throughout the genome. DNA ends are then ligated under dilute conditions in order to promote the formation of DNA junctions between the different strands of DNA linked through a protein complex. The crosslinks are then removed, and the DNA purified, before the annealing of custom 5C primers to individual junctions. A pool of 5C primers is used, represented by the bent lines. Forward primers possess a T7 adaptor (dark green segment), while reverse primers possess a T3c adaptor (purple segment) and a 5' phosphate. All primers have another segment that will bind complementary DNA immediately next to junction sites. Pictured annealing to the single stranded DNA are the red (forward) and blue (reverse) 5C primers. Only primers that are annealed to DNA, are immediately adjacent to one another, and possess a 5' phosphate on the reverse primer will then be ligated by Taq ligase. PCR amplification and labeling is done using the T7 and T3c adaptor sequences, and the resulting library of amplified 5C contacts is hybridized to a custom microarray for detection.

To perform a 5C experiment, a 3C library is first generated. 3C library preparation has been described in detail elsewhere [22]. Briefly, 3C libraries are produced by chemically fixing cells with formaldehyde to lock protein-protein and protein-DNA interactions in vivo (see Figure 1). A restriction enzyme is then used to digest the chromatin at specific sites across the genome. Samples are next diluted before the ligation step, such that ligation products are more likely to occur between DNA molecules bound together by protein complexes. The libraries are finally purified by proteinase K digestion and phenol-chloroform extraction. Resulting 3C libraries thus contain the entire genome's worth of unique ligation products whose relative levels are inversely correlated to the three-dimensional distance *in vivo *between restriction fragments in vivo.

5C quantifies 3C ligation products using a modified Ligation-Mediated Amplification (LMA) approach, which has also been described in detail elsewhere [25]. Briefly, 3C ligation products are detected with specially designed 5C primers that are complementary to the region(s) of interest and lie immediately upstream of the predicted 3C ligation junctions. Taq DNA ligase is then added to specifically ligate 5C primers at the junction of 3C products. LMA reactions can be performed at high level of multiplexing such that hundreds of primers could potentially be used in a single experiment to measure thousands of predicted chromatin contacts simultaneously. Universal tail sequences, located at the end of 5C primers, are then used to amplify and fluorescently label the library of synthetic 5C ligation products in a single PCR step. The labeled products are finally hybridized to custom microarrays for quantification. Conversion of microarray fluorescence intensities data obtained from a 5C experiment to interaction frequencies (IF) data can be performed using the *IFCalculator *program as described in [19,40]. Briefly, *IFCalculator *starts by excluding probes with intensity signals close to background and then combines the background-subtracted intensities of the remaining probes for the same fragment pair to obtain IF values and their standard deviations.

Hi-C data is generated in a similar manner to 5C data and was first described in Lieberman-Aiden *et al*. [18]. The technique includes additional steps of biotin fill-in and shearing before pull-down and paired-end sequencing. Hi-C can thereby be performed on a genome-wide scale and obviates the need for designing specific probes for each predicted pairwise junction. Its main drawback is the depth of sequencing required to obtain a good resolution at the IF level.

Although these assays are typically performed on diploid cells, the intra-chromosomal contacts found by both the 5C and Hi-C technologies can be treated as occurring within one homolog, as it has been previously shown that homologous copies of each chromosome occupy distinct nuclear positioning [41,42].

## Methods

In this section, we describe a probabilistic model of 5C/Hi-C interaction frequency data and the link between that data and the underlying chromatin 3D conformation. We then describe a Markov chain Monte Carlo (MCMC) approach to generate a representative sample of structures based on the experimental 5C IF data.

### Modeling chromatin conformations and 5C/Hi-C data

We model a chromosome (or a region of a chromosome) as a continuous piece-wise linear curve in 3D, where restriction site *i *is located at position *S*(*i*) = (*S_x_*(*i*), *S_y_*(*i*), *S_z_*(*i*)). The set of fragment end positions **S **= *S*(1), *S*(2),...,*S*(*n*), where *n *is the number of restriction sites considered, constitutes the conformation of the genomic region. In order to remain as general as possible and avoid introducing biases, we place no constraint on **S**. However, we discuss below how various types of priors or constraints could be used.

Pairs of fragments that are spatially close to each other generate large IF values while pairs of fragments that are spatially far from each other generate small IF values. We assume that the theoretical interaction frequency between fragment *i *and *j*, denoted *IF*(*i*, *j*), is inversely correlated with the distance between the two fragments in the 3D conformation: *IF*(*i*, *j*) = *f *(*D*_s _(*i*, *j*)), where *D*_s _(*i*, *j*) is the euclidean distance between restriction sites *i *and *j *in **S**, and *f*(·)is an appropriately chosen function of the form

(1) $$f\left(D_{S}\left(i,j\right)\right)\propto 1∕D_{S}{\left(i,j\right)}^{\alpha },$$

for some value of *α*. The choice of the value of *α *is discussed in Results.

Our experimental data consists of a set of observed pairwise interaction frequencies $\hat{IF}\left(i,j\right)$, measured by hybridization to a microarray or by sequencing. Because of noise in the measurements, $\hat{IF}\left(i,j\right)$ may not equal *IF*(*i*, *j*). Instead, we assume that $\hat{IF}\left(i,j\right)$ is a random variable whose distribution depends on *IF*(*i*, *j*). In the case of 5C data, we assume that the noise is independently and normally distributed, with a fragment pair specific standard deviation *σ*(*i*, *j*) obtained from the data using *IFCalculator*, as described in [40]. Then,

$$\begin{array}{c} \mathrm{Pr}\left[\hat{IF}\left(i,j\right)|IF\left(i,j\right),\sigma \left(i,j\right)\right]=\\ \;N\left(\hat{IF}\left(i,j\right);IF\left(i,j\right),\sigma {\left(i,j\right)}^{2}\right), \end{array}$$

where *N *(*x*; *μ*, *σ*^2^) is the normal density function.

Hi-C data is generated in a similar manner as 5C data, with the main difference being that ligation products are quantified by sequencing rather than hybridization. The observed read count *r*(*i*, *j*) for fragment pair (*i*, *j*), which is the quantity analogous to *IF*(*i*, *j*) in 5C experiments, is assumed to be dependent on the physical distance *D***_S_**(*i*, *j*) in the same manner as in 5C experiments. Although Hi-C read counts are not accompanied by noise estimates, they can be modeled by a binomial probability distribution, as suggested by Duan *et al*. [27], with *p*(*i*, *j*) ~ *r*(*i*, *j*)/∑_*a,b *_*r*(*a*, *b*), which we approximate, for computational efficiency reasons, using a normal distribution with variance equal to the mean plus a small constant:

(2) $$\mathrm{Pr}\left[\hat{r}\left(i,j\right)|r\left(i,j\right)\right]=N\left(\hat{r}\left(i,j\right);r\left(i,j\right),r\left(i,j\right)+\kappa \right).$$

The role of κ, which we set to 10, is to avoid having small read counts being assigned too low a variance.

The observed data $\hat{IF}$ defines a posterior distribution over the set of possible conformations of the chromatin: $\mathrm{Pr}\left[S|\hat{IF}\right]=\mathrm{Pr}\left[\hat{IF}|S\right]\cdot \mathrm{Pr}\left[S\right]∕\mathrm{Pr}\left[\hat{IF}\right]$. Since there are no constraints imposed on the structure space and the probability of the observed data $\left(\hat{IF}\right)$ is constant with respect to **S**, we get $\mathrm{Pr}\left[S|\hat{IF}\right]=\zeta \cdot \mathrm{Pr}\left[\hat{IF}|S\right]$, for some constant *ζ*, and thus

Pr[S|IF^]=ζ⋅∏i,j Pr[IF^(i,j)|IF(i,j)=f(DS(i,j),σ(i,j))].

This defines the posterior probability distribution over the space of structures, conditional on the observed IF data. A gradient descent approach, similar to that presented in *5C3D *by [19], could be used to identify locally optimal structures. However, there are often several different structures that fit the data almost equally well, so a probabilistic sampling approach that produces an ensemble of possible structures is advantageous.

### Sampling conformations from the posterior distribution

The Markov chain Monte Carlo (MCMC) algorithm is a method used to sample from a complex distribution (in this instance, from the posterior distribution of **S **given $\hat{IF}$), resulting in an ensemble of solutions *X*_1_, *X*_2_,..., *X_N _*[43]. Sampling from the posterior distribution consists of selecting an ensemble of conformations, where each conformation is selected with probability equal to its posterior probability. This is in contrast with maximum likelihood approaches, that seek to identify the (usually unique) structure S^* ^with the highest likelihood given the observed data. Usually the structure with the highest likelihood in our ensemble is a good approximation to S^*^, but the ensemble allows a much deeper understanding of the structure of the solution space. This sampling is performed using the Metropolis-Hastings algorithm [44]. A random structure *R*_0 _is initially chosen to seed the process (*t *= 0), where each point is placed randomly in a cube of side length $10\cdot avg\left(f\left(\hat{IF}\right)\right)$. We then iterate the following procedure. The current structure *R_t _*is randomly perturbed (see below) to obtain a new structure $R_{t}^{\prime }$. The posterior probability of the two structures are then compared. If $\mathrm{Pr}\left[\overset{\prime }{\underset{t}{R}}|\hat{IF}\right]>\mathrm{Pr}\left[R_{t}|\hat{IF}\right]$, the perturbation is retained and we set $R_{t+1}=R_{t}^{\prime }$. Otherwise, we retain $R_{t+1}=R_{t}^{\prime }$ with probability $\mathrm{Pr}\left[\overset{\prime }{\underset{t}{R}}|\hat{IF}\right]\text{/}\mathrm{Pr}\left[R_{t}|\hat{IF}\right]$, but set *R*_*t*+1 _= *R_t _*otherwise. Torrie and Valleau [43] showed that for values of *t *sufficiently large, $\mathrm{Pr}\left[R_{t}=S\right]=\mathrm{Pr}\left[S|\hat{IF}\right]$ and thus that the structures sampled are representative of the true posterior distribution. The period required for the Markov process to mix, known as the *burn-in *period, depends on the problem size and the type of perturbation performed.

The choice of the type of random perturbation to be performed can have a major impact on the length of the burn-in period. Perturbations must allow a quick and complete exploration of the conformation space, while only modifying the current conformation in a local manner. In addition, it is beneficial if the likelihoods of the new and old structures can be computed and compared quickly. In the context of protein structure prediction, the most commonly used approach is to randomly modify one of the bond angles between consecutive amino acids. Although this approach is in principle applicable to our type of data, it would yield poor results, as a large number of pairwise distances would be significantly modified by any angular change. Instead, we elected to perturb structures by randomly choosing one point *S*(*i*) along the structure and moving it by a vector $\vec{v}$ randomly chosen within a sphere of radius *r *(manual investigation showed that *r *= 0.25 nm yields good results for both 5C and Hi-C data). Clearly this type of perturbation allows the exploration of the full structure space from any starting configuration. The likelihood of the resulting structure is then quickly obtained from that of the old by updating the terms corresponding to the pairs of points involving *i*.

#### Assessing Mixing

During the first iterations of the MCMC sampling process, called the burn-in phase, structures *R*_1_,...,*R_k _*are highly dependent on *R*_0_, the initial structure, and do not represent a proper sample in our conditional probability distribution. It is critical to be able to determine at what point *m *the Markov process has mixed, i.e. for what value of *m *is *R_m _*essentially independent of *R*_0_. After mixing, i.e. for *k *≥ *m*, any sample *R_k _*is representative of the target distribution. Furthermore, for *δ *sufficiently large, samples *R_k _*and *R*_*k *+ *δ *_are independent.

Several approaches exist to determine when a Markov chain has mixed, and what value of *δ *is suitable. The standard approach is to compare the probability distributions over the state space obtained from parallel runs started from different initial conformations, and keep sampling until the two become indistinguishable. Because our state space is continuous and high-dimensional (3 *n *parameters), no structure is actually ever sampled more than once, making this approach unusable. A literature search did not yield a ready-made solution for assessing the convergence of MCMC for structural inference, so we generalize the standard approach as follows. We run two independent chains *R *and *R' *in parallel, from independently chosen initial conformations *R*_0 _and $R_{0}^{\prime }$. After *k *iterations, we say that mixing is achieved if the samples $R_{k}=\left\{R_{k∕2},R_{11k∕20},...,R_{k}\right\}$ and $R_{k}^{\prime }=\left\{R_{k∕2}^{\prime },R_{11k∕20}^{\prime },...,R_{k}^{\prime }\right\}$ cannot be distinguished from each other. Specifically, the average pairwise structural distances (see below) among structures in $R_{k}$ is compared to the average pairwise distances between pairs of conformations from $R_{k}\times R_{k}^{\prime }$. If the two means are within 10% of each other, we conclude that mixing is achieved and start collecting samples every *δ *= *k*/20 iterations: *X*_1 _= *R_k_*, *X*_2 _= *R*_*k *+ *δ*_, *X*_3 _= *R*_*k *+ 2_·*δ*,...,*X_N _*= *R*_*k *+ (*N *- 1)_·_δ_. This not only ensures that mixing has occurred, but also that subsequent samples, taken every *δ *iterations, are essentially independent.

### Clustering of structure ensemble

The set of structures *X*_1_, *X*_2_,..., *X_N _*sampled by the *MCMC5C *program is representative of the distribution of structures that fit the observed interaction frequency data. In several cases, it can be useful to cluster structures from this ensemble based on their similarity, for example to identify subfamilies of structures whose properties can be assessed and contrasted. In addition, when ensembles from parallel runs are obtained, mixing can be assessed by verifying whether structures from each run cluster together (in which case mixing is not achieved) or not. Finally, ensembles from MCMC runs executed on different datasets can reveal similarly/dissimilarity between chromatin conformations under different conditions.

We first define a measure of distance between two structures and then use hierarchical clustering (Ward's method) [45] to identify groups of similar structures. A measure of similarity between structures that is commonly used in the area of protein and RNA structure prediction is the root-mean squared deviation (RMSD), which requires first aligning (through rotations and translations) the two structures being compared, and then summing the square of the distances between corresponding points along the structure [46,47]. Although applicable to our structures, we prefer a simpler approach that has the advantage of not requiring an alignment of the structures (it is rotationally, translationally, and reflectionally invariant) while being more flexible in the type of geometric similarities it can capture. We first define the *N *× *N *intra-structure distance matrix *D***_S _**as the matrix of geometric Euclidean distances between each pair of points *i*, *j *in structure **S**: The distance *dist*(**S**, **T**) between structures **S **and **T **is then:

$$dist\left(S,T\right)=\sqrt{\underset{i.j}{\sum }{\left(D_{S}\left(i,j\right)-D_{T}\left(i,j\right)\right)}^{2}}.$$

Note that two structures that are mirror images of each other will have distance zero. Indeed, such structures cannot be distinguished based on 5C/Hi-C data. The structures from an ensemble *X*_0_, *X*_1_,..., *X_N _*are clustered by first computing *dist*(*X_i_*, *X_j_*) for all 1 ≤ *i *≤ *j *≤ *N *and then using Ward hierarchical clustering [45]. This clustering is used to determine the existence and number of structure subfamilies and the members of each subfamily. Visualization is accomplished with both a hierarchical tree dendrogram and a heatmap representation. Visual inspection is performed to determine the tree height cutoff and number of subfamilies and for each subfamily the member structure with the highest posterior probability is chosen as the representative structure for that cluster. Choosing the maximum likelihood structure from each cluster as representative and assigning it a weight proportional to the number of the structures in its cluster allows focusing on a small number of representative structures.

### Identification of reliable substructures

The ensemble of structures generated by the *MCMC5C *program will typically contain substructures that are highly constrained by the $\hat{IF}$ data and are thus present in the vast majority of structures, and others that are highly variable. Knowing what aspects of the reported structure are reliable is critical to guide downstream experimental validation. While this can sometimes be done by visual inspection of the superimposition of the structures from the sample, a more automated approach is usually desirable. This can be achieved by identifying a subset of *k *fragments whose pairwise distances are best conserved across the structures in the ensemble. To this end, we first compute the standard deviation *s*(*i*, *j*) of the intra-structure pairwise distance for each pair of points *i *and *j*, across all samples from the ensemble. We then identify the set of *k *fragments with the smallest total pairwise standard deviation using a greedy algorithm.

### Measuring structural properties

**Figure 2:**
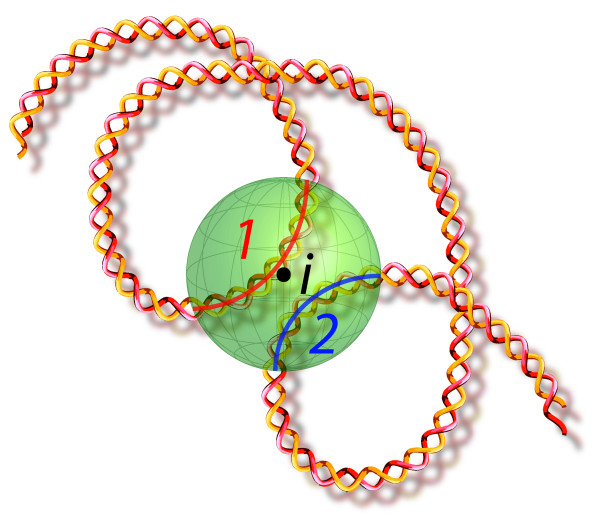
**Structural Properties**. Schematic diagram of Structural Properties. The shaded sphere with radius *r *is centered at base *i*. The nucleotides that lie within the sphere and delineate compartment 1 (nucleotides consecutive to base *i *before leaving sphere, indicated with a red arc) are counted as the base condensation measure and the nucleotides that lie within the sphere and delineate compartment 2 (nucleotides on sequence that has exited and re-entered sphere, indicated with a blue arc) are counted as the base looping measure. The total number of nucleotides contained within the sphere is counted as the base density measure.

One of the key advantages of a sampling approach, compared to non-probabilistic or maximum likelihood approaches, is its ability to estimate the distribution of various structural properties, and thus to report both averages and confidence intervals for the selected properties. This is particularly useful when aspects of the conformation of chromatin remain poorly determined by the data; a researcher needs to know to what extent a particular structural property of interest is observed in just a single solution (e.g. the maximum likelihood solution) or present in all (or most) possible structures. To this end, *MCMC5C *allows the easy estimation of the distribution of various structural properties. Here, we focus on three properties of interest (local base density, condensation, and looping), that are evaluated for every position *i *along the region of interest (see Figure 2). Local density at position *i *along the sequence is the number of DNA bases located within a sphere of radius *r *centered at position *i*. The local base density can be decomposed into two terms: compaction and looping. Compaction measures the number of DNA bases located within the sphere and *consecutive to position i*, whereas looping counts the number of bases inside the sphere but outside the portion containing *i*.

## Results and Discussion

### 5C datasets

Our modeling approaches were applied to three sets of 5C data studying the chromatin structure of the HoxA cluster (see Dostie *et al*. [25] and Figure 1 for a summary of the 5C procedure). The first pair of experiments (previously published in [19]) studies the conformation of the HoxA cluster during THP-1 cell differentiation from myelomonocyte to macrophage. 5C libraries were produced in both the undifferentiated myelomonocyte state and in the differentiated macrophage state (96 hours after treatment with phorbol myristate acetate (PMA)). For the third set of experiments (unpublished data), 5C data was generated for the same genomic region in a MLL-ENL fusion cell line (HB-1119) that expresses a different MLL-fusion protein than the THP-1 cell line and induces aberrant over-expression of the 5' HoxA genes [48,49]. In both datasets, the genomic region analyzed spans 142 kb and contains 11 protein coding genes. The region contains 42 restriction sites for the BglII restriction enzyme, which was used for the experiment. Each 5C library was hybridized onto a custom array with a set of probes corresponding to every potential pair of fragments (due to the forward and reverse primer design used, only interaction frequencies between an even numbered and an odd numbered fragment are measured). The set of probe intensities were normalized using corresponding gene desert regions as previously described in Fraser *et al*. [19] and analyzed using the *IFCalculator *program [19,40] to perform outlier detection and obtain interaction frequency and standard deviation estimates for every fragment pair considered. Although nearby sites along the sequence have elevated interaction frequencies, IFs between pairs of fragments located more than 10 kb are generally close to background levels, with several notable exceptions likely resulting from chromatin looping (see Additional File 1).

### Choice of distance-to-IF transformation

Although it is clear that pairwise interaction frequencies are inversely correlated with the physical distance between any pair of fragments in the chromatin conformation [18,22], there is no consensus on how IF depends on physical distance. Duan *et al*. [27] perform distance-to-IF conversions by first considering only short-range interactions (involving pairs of points that are close together along the sequence) and obtaining physical distances for these pairs based on polymer models. A given long-range IF value is then mapped to the polymer-based distance that is the most likely to have resulted in that value. The resulting conversion approximately follows *d *∝ 1/*IF*. Mateos-Langerak *et al*. [50] also suggest a relationship of the form *d *∝ *IF^α^*. Bau *et al*. [28] convert their IF via a linear transformation of the IF's z-score. Tanizawa *et al*. [29] relate IF to physical distance by using a loess regression on a set of physical distances measured by 3D-FISH, but do not report the parameters of this regression. The extent to which the function mapping IF values to physical distance depends on the specific experimental protocol remains unclear.

In the absence of independent structural measurements for the HoxA cluster, we argue that the most accurate model is the one that is best able to predict unseen pairwise interaction frequencies. For each of a set of possible values of *α *in *d *= *C*/*IF^α^*, a leave-one-out cross-validation (LOOCV) experiment was performed, excluding in turn the interaction frequency measurement of each pair of points, *n*, inferring a maximum-likelihood structure from the remaining data points, and comparing the left-out IF value to the theoretical IF value given the distance between fragments *i *and *j *in the obtained structure. Specifically, let $S_{\left(i,j\right);\alpha }^{*}$ be the maximum likelihood structure found by *MCMC5C *on a data sets consisting of the IF values for all fragments pairs *except *(*i*, *j*), when using value *α *to transform physical distance to interaction frequencies. We then define

$$MSE(\alpha )=\frac{1}{n}\sum_{\left(i,j\right)}{\left(D_{S_{(i,j);\alpha }^{*}}{\left(i,j\right)}^{-\alpha }-I\hat{F(i,}j)\right)}^{2}.$$

**Figure 3:**
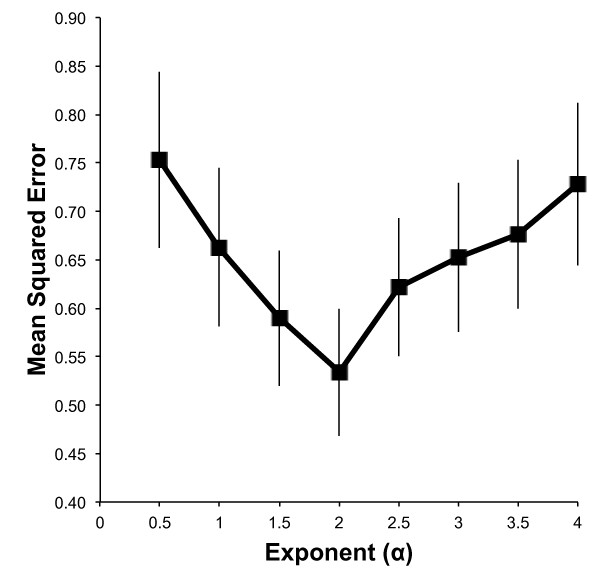
**Leave-one-out cross-validation**. Value of the mean-squared-errors as a function of *α*, obtained for a leave-one-out cross-validation on the HB-1119 dataset. The minimum error is found for an exponent of 2.0, although values of *α *between 1 and 3 do not produce significantly worse errors.

Figure 3 shows the value of the *MSE *for different values of *α*, for the HB1119 dataset. A minimum is reached at *α *= 2.0, which is the value we retain for the rest of this study, but values of *α *between 1 and 3 cannot be rejected. Similar results are obtained on the THP-1 5C data sets, although with a larger overlap between confidence intervals. We add that an alternate approach, which posits that the ideal choice of *α *is that which maximizes the likelihood of the maximum likelihood structure found, suggests similar values for *α *(data not shown). Without physical measurement of the distance between pairs of points along the sequence, it is difficult to accurately estimate the value of *C*. However, based on the average IF value of pairs of fragments located less than 5kb apart along the sequence and following Bystricky *et al*. [51] that packed chromatin has a physical length of 1 nm for every 110-150bp, *C *was estimated as approximately 50 nm.

### Mixing and convergence

**Figure 4:**
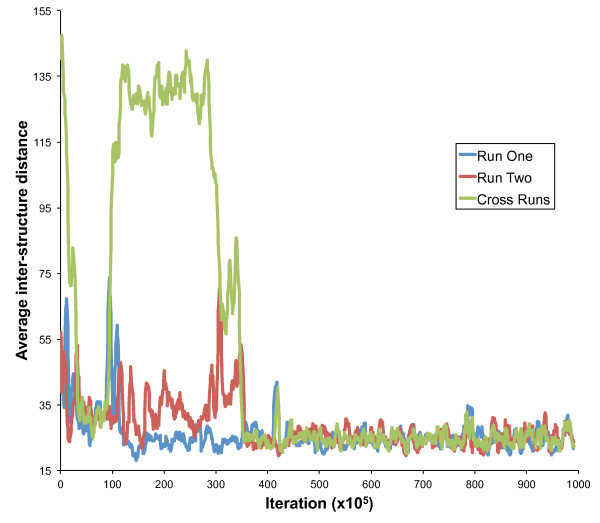
**Mixing of parallel *MCMC5C *runs (HB-1119 dataset)**. Distance between consecutive structures (sampled every 10^6 ^iterations) from within one of two parallel *MCMC5C *runs (blue and red curves) or across the two runs (green curve), on the HB-1119 5C dataset. The runs converge to the same distribution very rapidly (in less than 250 seconds) and the cross-run distance (green) drops to within the same range as the within-run distances (blue and red curves) after 350 × 10^5 ^iterations.

**Figure 5:**
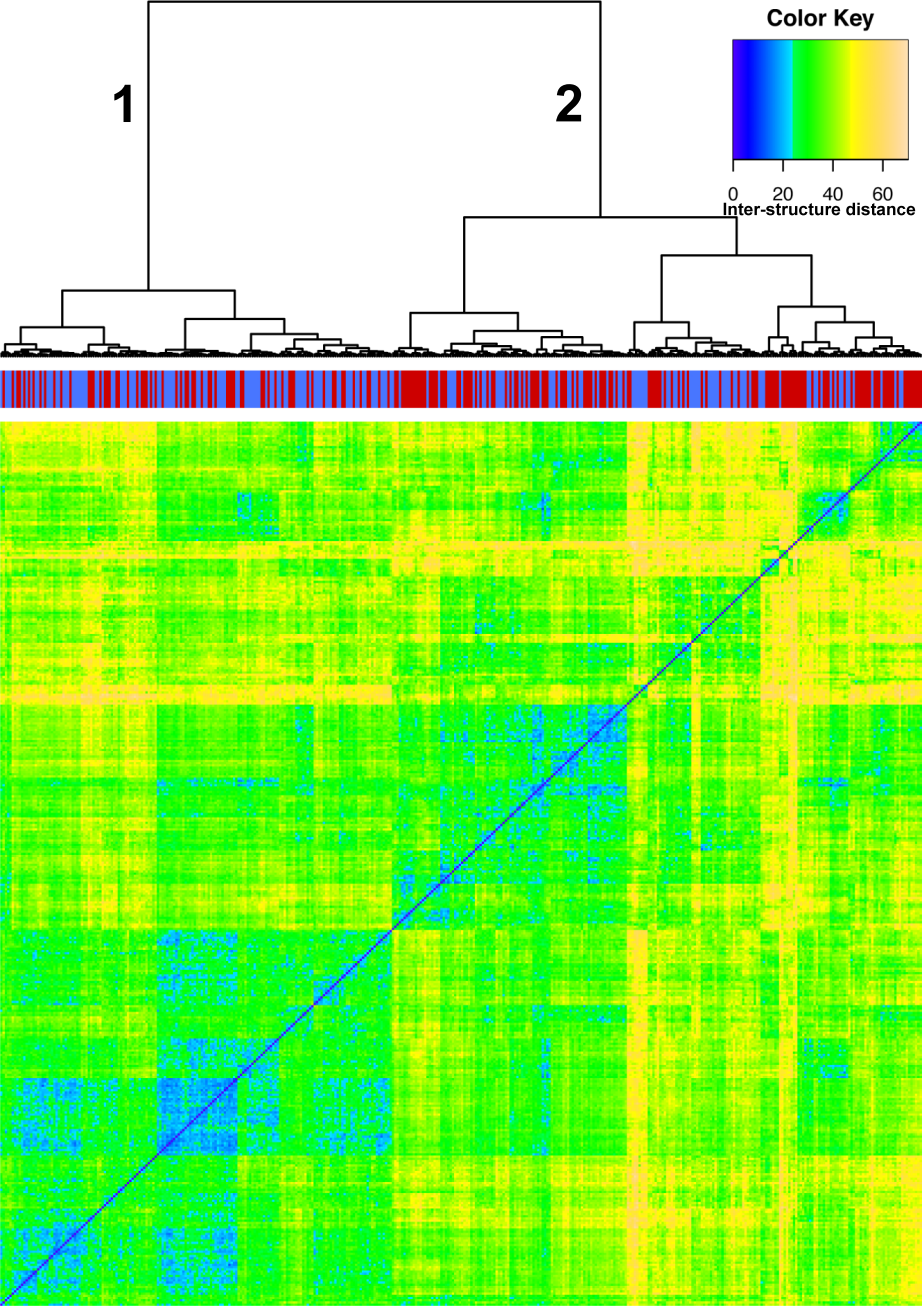
**Mixing and subclustering of HB-1119 structures**. Mixing and hierarchical clustering (Ward's method) of structure similarity. The five-hundred structures come from two parallel *MCMC5C *runs on the HB-1119 dataset (pools of 250 structures from each run were used). The colors along the top indicate which run each structure originated from (run one = blue, run two = red) and demonstrates that the sampling process has successfully mixed. The blocks in the heatmap and the dendrogram indicate the presence of sub-clusters of structures (numbered in the dendrogram). The two clusters (numbered 1 and 2) both contain structures from the two parallel runs (blue and red vertical bars), indicating that the structures are conserved across runs and are not an artifact of the burn-in process.

The convergence of the MCMC sampling procedure was tested on all datasets, but for simplicity we focus on those obtained on the HB-1119 5C data set. We first studied how long a burn-in phase is required before parallel runs converge to a similar conformation distribution (see Methods). Figure 4 shows that mixing is achieved after approximately 350 × 10^5 ^iterations, which requires less than 250 seconds of running time. Passed this point, structures sampled every 10^6 ^steps from the two parallel runs are undistinguishable from each other and sample structures from the same distribution. 250 structures were sampled after burn-in from each of the two runs. The two ensembles of structures were then combined and the 500 structures were clustered based on their structural similarity (see Figure 5 and Methods). We observe that structures from the two runs are interleaved in the clustering, confirming that both runs are correctly sampling from the same posterior distribution. Analysis of the two THP-1 5C datasets produced similar results, and runs of a larger number of parallel MCMC chains confirm that they all sample similar structures.

Additional File 2 compares the likelihood of the structures sampled by *MCMC5C *to those found by several runs of the gradient descent program *5C3D*, started from different initial structures. Although both approaches succeed at identifying credible structures, we observe that the structures found by *5C3D *generally have lower likelihoods than those sampled by *MCMC5C *- indeed, the misfit function optimized by *5C3D *is not equivalent to the likelihood function, which explains the slight decrease in likelihoods observed for many *5C3D *runs past a certain number of iterations. Importantly, the five *5C3D *runs converge to three different solutions, hinting that this type of approach is subject to getting stuck in local optima.

### Accuracy of structure predictions on simulated data

**Figure 6:**
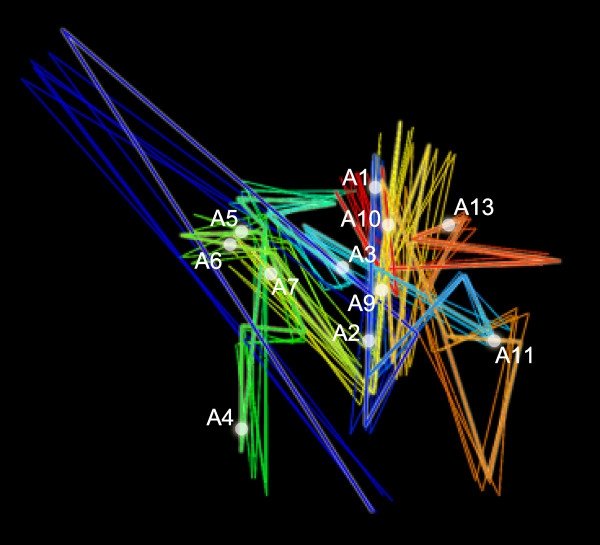
**HB-1119 Structures from simulated data aligned to gold standard structure**. The ''gold standard'' structure is used as a reference structure to which structures from four different parallel *MCMC5C *runs on simulated data generated from the gold standard structure are aligned. The gold standard structure is shown highlighted with a white glow and the transcription start sites for the HoxA genes are annotated. The structures found from the simulated data are shown in superimposition to the gold standard structure and show a high degree of alignment.

Having shown proper mixing of the sampling process, we then asked whether the structures produced faithfully correspond to the true structure. In the absence of external experimental data at the appropriate resolution, we used simulated data. Starting from a known "true" structure, we generated the corresponding simulated IF data (with noise), and assess our ability to recover the initial structure. Using the HB-1119 5C dataset, we sampled the structure with the highest posterior probability using *MCMC5C*. This structure was then used as a ''gold standard'' from which simulated noisy IF data was generated, based on the noise model described above. Four parallel runs of *MCMC5C *were then performed (from different random initial structures) on the simulated dataset and the structures with the highest posterior probability structure from each run were aligned to the original gold standard structure (Figure 6 and Additional Files 3 and 4). Clearly, *MCMC5C *was able to sample structures from the posterior distribution defined by the interaction frequency data by recovering structures that closely match the gold standard from which the simulated interaction frequency dataset was generated.These results suggest that the sampling approach succeeds at finding the correct structure, at least under the assumption that the IF data is generated from the pairwise distances using our model.

Interestingly, the set of four maximum likelihood structures found by the four parallel *MCMC5C *runs actually contained topological mirror-images of otherwise nearly identical structures. These ''enantiomer'' structures have equal probability given our model of IF data and the structures were mirrored as required before the superimposition shown in Figure 6 was performed.

### Clustering of conformational ensemble

Upon analysis of the mixing between two parallel *MCMC5C *runs on the HB-1119 5C dataset, we observed two distinct clusters in the heatmap (see Figure 5) that correspond to two subclasses of structures, each of which is sampled from both of the two parallel runs (seen by the mixing of the blue (run one) and red (run two) labels at the top of the heatmap). The clusters obtained are robust to changes in the clustering algorithm: the cluster membership determined by the hierarchical Ward clustering algorithm agrees at 85% with that obtained by the k-means algorithm, which operates in structure space rather than based on a distance matrix, suggesting that the two main clusters are indeed distinct and well separated. We note that we do not necessarily expect these two clusters to reflect two different chromatin conformations present in the population of cells used to generate the 3C library. Instead, they represent two possible conformations for the population-wide average conformation.

The posterior probability of each class can be estimated as the fraction of the samples belonging to it. The two largest clusters, whose structures mainly differ in the position of the loop in the region lying between the HoxA11 and HoxA13 genes, account for 42% and 58% of the structures sampled (these two main classes are not the two enantiomers discussed above - indeed, because of our structure similarity measure, enantiomers are considered as identical). This finding illustrates one of the benefits of *MCMC5C *over *5C3D *by demonstrating the ability to discover different subclasses of structures that fit the experimental data almost equally well.

### Analysis of HoxA conformational ensembles

**Figure 7:**
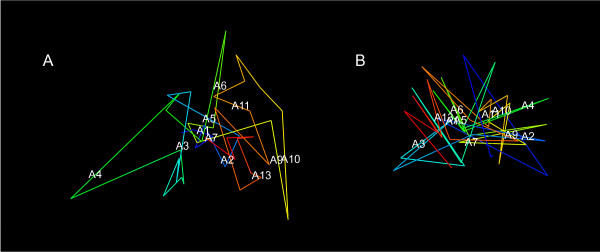
**Models of HoxA cluster before and after differentiation**. Maximum likelihood structures found by *MCMC5C *from the undifferentiated and differentiated THP-1 datasets (A and B, respectively). The HoxA gene transcription start sites are annotated on each of the structures.

Figure 7 A and B shows structures obtained by *MCMC5C *on the undifferentiated and differentiated THP-1 5C datasets (ensembles of 500 structures were sampled from runs consisting of 5 × 10^9 ^iterations). Visual inspection reveals regions looping out of the core structure in the undifferentiated state, such as the regions shown in green and in yellow, corresponding to the genomic region that includes the HoxA9 and HoxA10 gene transcription start sites (see Additional Files 5, 6, 7, and 8 for movies showing the rotating 3D structures and PDB files for each state). Contrasting the ensembles obtained in undifferentiated and differentiated conditions, it is readily apparent that upon differentiation the structure adopts a more compact form that occupies a smaller volume. The regions that are seen to be extruded in the undifferentiated state are collapsed into the core of the structure in the differentiated state. These results agree with those previously shown by Fraser *et al*. [19] whereby the tight packing of the chromatin in the differentiated state correlates with an experimentally measured decrease in HoxA gene expression (HoxA9, A10, A11, and A13) upon differentiation.

**Figure 8:**
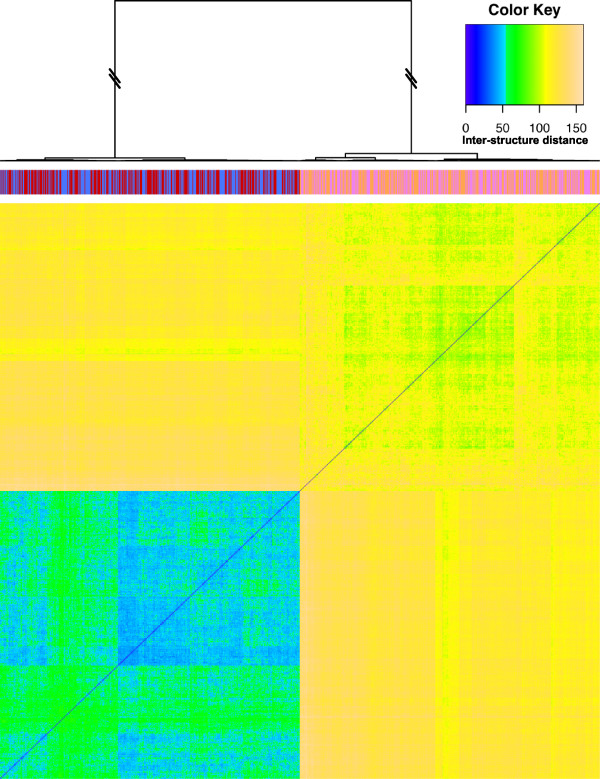
**THP-1 clustering of undifferentiated and differentiated structures**. Hierarchical clustering (Ward's method) of one-thousand structures from four parallel *MCMC5C *runs, two on the undifferentiated THP-1 dataset and two on the differentiated THP-1 dataset (250 structures each). The colors along the top indicate which state each structure originated from (undifferentiated run one = blue, run two = red; differentiated run one = pink, run two = orange) and demonstrate a clear distinction between the two states, indicating that the undifferentiated and differentiated cell states specify different structure signatures.

Figure 8 shows a clustering of the pooled ensembles obtained from the THP-1 undifferentiated and differentiated states. The samples from each of the two datasets form two very distinct clusters, although there is clearly variability within each group. This supports previous observations that the HoxA cluster undergoes a major conformational change upon differentiation of THP-1 cells [52] but confirms for the first time that the observed differences are not simply due to uncertainty in the exact conformation under each condition. The two clusters exactly capture all of the structures corresponding to each of the states in two distinct clusters, supporting our findings from the visual inspection of the structures and suggesting a different Chromatin Conformation Signature (CCS) for each of the states. However, biological replicates of each 5C experiment will be required to determine whether the observed differences stand out above inter-experiment variability.

While visualization is a powerful analysis approach, chromatin regions whose structure is well supported by the 5C data are better identified by our reliable subset identification algorithm, which identifies, from a given ensemble of structures, the subset of fragments whose spatial relationship varies the least within the ensemble. The subset of fragments that are the most conserved across the ensemble of structures (see Additional File 9) are found to lie within the central core region of the structures. These fragments are spatially close to each other and may be involved in looping contacts that are important for the maintenance of the chromatin structure and are therefore highly conserved. These results are observed in the ensembles of structures for both of the cellular states, whereby the most conserved substructures are found to lie within the regions corresponding to the strongest contact points.

#### Estimation of structural properties

**Figure 9:**
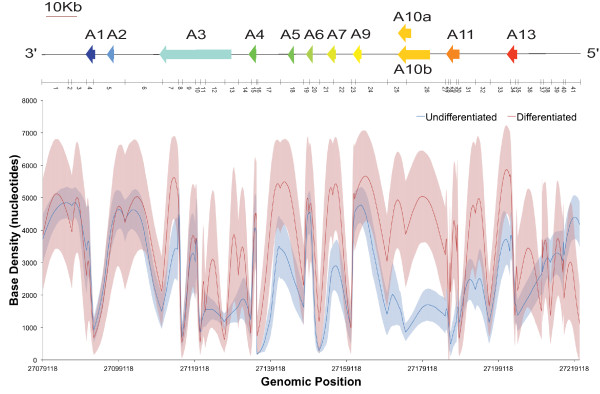
**Base Density analysis of undifferentiated and differentiated THP-1 cells**. Analysis of base density comparing the undifferentiated (red curve) and differentiated (blue curve) cell states. The genes in the HoxA locus are shown aligned above the plot. A pool of one-hundred structures generated by *MCMC5C *were used for each state. The base density measure was calculated with a sphere of radius one (1.0) every tenth base. The error bars report the standard deviation.

A powerful use of *MCMC5C *is in the discovery of structural properties that are strongly supported by the 5C data. This allows researchers to formulate solid hypotheses while avoiding relying on properties that may only be present in a handful of possible structures. It is straightforward for researchers to implement new modules in *MCMC5C *that will evaluate the structural properties of their choice. Here, we utilize this functionality to assess and contrast the degree of looping (long-range chromatin contacts) and base density (see Methods and Figure 2) along the HoxA cluster, in undifferentiated and differentiated THP-1 cells. The mean base density value and its standard deviation across the ensemble of structures are reported in Figure 9. We previously showed that expression of Hox genes located at the 5' end of the HoxA cluster undergo repression upon terminal differentiation in Fraser *et al*. [19]. This region includes the HoxA9 and HoxA10 genes that have been shown to be oncogenic and are induced by the aberrant expression of the MLL-AF9 translocation protein present in the THP-1 cell line [52]. Analysis of the local base density reveals a significant increase in base density corresponding to the region of the HoxA cluster containing the genes that are repressed upon differentiation. Further analysis of the base looping measure (see Additional File 10) reveals the creation of a looping contact in this same region upon differentiation and repression of gene expression. These observations fit with previous findings that repressed genes reside in condensed heterochromatin and suggest a model of gene repression during differentiation that involves the formation of a looping contact that serves to close the chromatin structure of the HoxA cluster to aid in repressing (or maintaining a repressed state) of the genes located in that region, and warrants further investigation. Finally, we note that without the help of the base density confidence intervals obtained from our structure ensemble, it would have been tempting to interpret many of the apparently large differences between mean base densities as potentially biologically meaningful. However, those differences are not statistically significant, as the corresponding confidence intervals, whose size are quite variable along the sequence, overlap in these regions.

### Analysis of a Hi-C dataset

**Figure 10:**
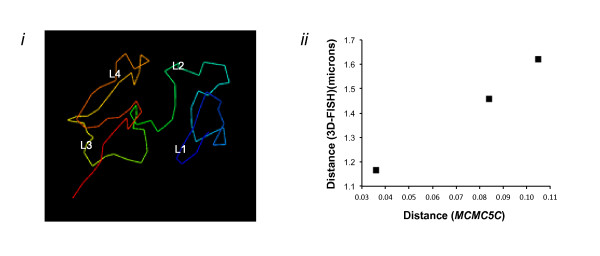
**Modeling of human chromosome 14**. (Left) *MCMC5C *model of human chromosome 14 from the Hi-C dataset. The midpoints of the 3D-FISH probes used by Lieberman-Aiden *et al*. [18] are annotated as L1, L2, L3, L4 and were designed such that in consecutive order the probes alternate between compartments. The structure adopts a loosely defined spiral form which brings the probes from within either compartment (A: L1 and L3, B: L2 and L4) in closer physical proximity than between pairs of probes across compartments. (Right) Distances inferred by *MCMC5C *correspond to physically-measured distances. X-axis: average Euclidean distance in the ensemble of 250 structures sampled by *MCMC5C*. Y-axis: median 3D-FISH physical distance measured by Lieberman-Aiden *et al*. [18]. Even though probe L3 is located between probes L2 and L4 in the linear sequence, probes L2-L4 are closer together in the model than L3-L2, indicating preferential organization of probes belonging to the same compartment (B) than across compartments (A-B) as initially reported in Lieberman-Aiden *et al*. [18].

To demonstrate the applicability of our method to other datasets, we chose to model the long arm of human chromosome 14 (88.4 Mb region) from Hi-C data published by Lieberman-Aiden *et al*. [18] at a 1Mb resolution (89 fragments in total). We generated an ensemble of 250 structures sampled over 5 × 10^10 ^iterations. Figure 10 (left) shows the maximum-likelihood structure found (see Additional File 11 for a better 3D view). Lieberman-Aiden *et al*. [18] proposed the existence of two physically disjoint compartments, whereby compartment A was found to correlate with open and actively transcribed chromatin, while compartment B was found to be more densely packed and repressed. The authors designed four 3D-FISH probes (termed L1, L2, L3, and L4) that lie consecutively along chromosome 14 but alternate between compartments (A: L1 and L3; B: L2 and L4) and showed that the non-consecutive regions of the chromosome that belong to the same compartment appear to be physically closer than those that do not [18]. Our results using *MCMC5C *weakly supports this hypothesis, with the 3D-FISH probes L2 and L4 indeed being in close proximity. Importantly, we used an ensemble of 250 structures to estimate the distribution of predicted Euclidean distances between each pair of probes and found an excellent linear correlation with the physical distances measured by Lieberman-Aiden *et al*. [18] using 3D-FISH (see Figure 10 (right). This suggests not only that our model may be physically realistic, at least at a broad level, but also that the IF-to-distance transformation used is appropriate.

### Implementation and running time

The *MCMC5C *program is implemented in Java and is available at http://Dostielab.biochem.mcgill.ca. The program takes the experimental interaction frequency data (with standard deviations) and the restriction enzyme genomic cut sites as input and produces an ensemble of structures as PDB files as output. Individual runs were performed on a 2.26 Ghz Intel Core 2 duo machine with 4 GB of RAM, while simultaneous parallel runs were performed on a cluster comprised of 20 Apple dual-processor 2.3-GHz compute-node G5s (2 GB of RAM each). Execution time increases with the number *n *of fragments in the structure. Each MCMC iteration runs in time *O*(*n*), as only the fragment pairs involving the fragment that was moved need updating. However execution time is mostly driven by the time to mixing, which not only depends on the size of the structure but also on how "unique" the solution is; a situation where the pool of likely structures is very small will lead to faster mixing than one where the set of possible solutions is much larger and involves many very different structures. Each of our 5C data sets consisted of 41 fragments, yielding between 335 and 398 interaction frequency measurements (the *IFCalculator *excluded some measurements because of their excessive variance between microarray probe replicates). Mixing was achieved in approximately 3.5 × 10^7 ^iterations for each data set, which took ~250 seconds. Ensembles of 1000 structures were then obtained by running the chain for approximately two hours. For the analysis of the Hi-C data from human chromosome 14, which consists of 89 fragments and 3916 IF pairs, mixing was achieved after 4 × 10^7 ^iterations (~800 seconds) and 250 structures were obtained in approximately 2.5 hours. However, our attempts to use *MCMC5C *on the full Hi-C dataset from Lieberman-Aiden *et al*. [18], consisting of data from all 23 human chromosomes, failed to achieve mixing after 24 hours of execution.

## Conclusions

The role of high-level chromatin conformation in regulating gene expression is now well accepted, although only a few loci have been studied in detail [17,20,53-55]. Chromosome conformation capture-based technologies (3C [22], 5C [56], Hi-C [18], and their variants [23,24,26,27,57]) offer the ability to measure properties of the high-level chromatin organization by measuring the interaction frequency between genomic fragments. The resolution and accuracy of these techniques is rapidly improving, and, with the use of next generation sequencing, their throughput is increasing while their cost is decreasing. For these reasons, these technologies are increasingly popular.

Whereas the technological advances allow increasingly complex assays to be performed, few computational and statistical tools exist to analyze the data resulting from such experiments, although good approaches exist to help design the experiments [18,19] or handle and visualize their output [17-19,26,27]. We previously developed the *5C3D *program, which aims at producing the best-fitting conformation for a given dataset. Similar optimization-based approaches have also been used to model the structure of the yeast genome [27,29], and the *α*-globin locus [17,28]. However, the absence of statistical or Bayesian approaches make it impossible to assess the reliability of the predicted conformation. Downstream analyses are thus limited to qualitative observations that may or may not be reliable. In this paper, we introduce a probabilistic framework to address this problem. By sampling from the posterior probability distribution over conformations, *MCMC5C *produces an ensemble of different structures that are possible given the data and can find subclasses of structures that fit the data equally well. Overlaying these conformations in a visualization tool such as *PyMOL *[58] readily allows the identification of reliable and less reliable aspects of the conformation. Using ensembles allows the discovery of subclasses of structures and the estimation of structural properties, together with their distribution, which allows the user to focus on statistically sound properties or differences between datasets. Although we acknowledge that more refined probabilistic models of 5C and Hi-C data will eventually be required to improve the accuracy of the structure predictions, those will be easily accommodated with *MCMC5C*.

None of the existing computational approaches to model 3D chromatin structures make use of advanced physical models of DNA and chromatin, although the approach of Duan *et al*. [27] uses a simple polymer physics model to transform interaction frequency, while Tanizawa *et al*.[29] include simple sets of constraints derived from polymer physics. The methodology described in this paper attempts to model chromatin without specifying any type of hard constraints on the predicted structure, although such constraints could easily be included if desired. Our probabilistic framework also allows for the easy integration of structure priors based on free energy. Although excellent models of polymers exist (e.g. Langowski and Heermann [59]), it is unclear to what extent these models are informative at the scale we are considering (average fragment size of 4 kb in the case of our 5C data and 1Mb in the case of the Hi-C data).

A number of interesting directions should be investigated in the future. Time to mixing remains the main obstacle to running *MCMC5C *on very large datasets such as the whole-genome Hi-C dataset of Lieberman-Aiden *et al*. [18]. We are currently working on considering other types of structural perturbations for the MCMC sampling, such as modifying the torsion of a given fragment or the angle between two fragments, or a combination of several types of perturbation. These advances should allow for more rapid sampling from the structure space, thereby aiding in the discovery of alternative conformations belonging to small subclusters of structures.

To conclude, we believe that probabilistic tools like *MCMC5C *are essential for the reliable analysis of data from the 3C-derived techniques such as 5C and Hi-C. By integrating complex, high-dimensional and noisy datasets into an easy to interpret ensemble of three-dimensional conformations, *MCMC5C *allows researchers to reliably interpret the result of their assay and contrast conformations under different conditions.

## URIs

*MCMC5C *is available at http://Dostielab.biochem.mcgill.ca. Detailed protocols, 3C and 5C support information (design and analysis) can also be found at this location.

## Authors' contributions

MB and MR designed the computational analysis methods that MR implemented. JF and MAF generated the 5C datasets with JD's supervision. All authors discussed the results. MR drafted the manuscript and all authors read, edited, and approved the final manuscript. MR, JD, and MB wrote the manuscript.

## Supplementary Material

Additional file 1 **Compaction profile of the HoxA region for THP-1 undifferentiated and differentiated cell states**. Compaction profile of the HoxA cluster for both the undifferentiated (blue squares) and differentiated (red diamonds) THP-1 cell states. The average interaction frequency value diminishes with increasing linear genomic distance between the fragment pair, but strong contacts can be seen to exist between fragments at distances over 10-kb apart. Click here for file

Additional file 2 **HB-1119 Likelihoods of *MCMC5C *and *5C3D *structures**. Likelihood of the structures produced by *MCMC5C *and by several runs of *5C3D*, as a function of the number of iterations (note the different scales of the x-axis for the two approaches). *5C3D *very quickly converges to locally optimal structures that are slightly sub-optimal, and different runs converge to different solutions. Click here for file

Additional file 3 **HB-1119 Structure alignment movie**. A QuickTime movie of the HB-1119 ''gold-standard'' structure aligned with the best structures from the four parallel *MCMC5C *runs on the simulated data. The reference structure is annotated with the transcription start sites for the HoxA genes. Click here for file

Additional file 4 **HB-1119 Ensemble**. A zip file containing the ensemble of PDB structures generated by *MCMC5C *from the HB-1119 5C dataset. Click here for file

Additional file 5 **5C HoxA cluster undifferentiated movie**. A QuickTime movie of the human HoxA cluster in the undifferentiated state as determined by *MCMC5C *from 5C data. Click here for file

Additional file 6 **5C HoxA cluster differentiated movie**. A QuickTime movie of the human HoxA cluster in the differentiated state as determined by *MCMC5C *from 5C data. Click here for file

Additional file 7 **THP-1 Undifferentiated ensemble**. A zip file containing the ensemble of PDB structures generated by *MCMC5C *from the THP-1 undifferentiated 5C dataset. Click here for file

Additional file 8 **THP-1 Differentiated ensemble**. A zip file containing the ensemble of PDB structures generated by *MCMC5C *from the THP-1 differentiated 5C dataset. Click here for file

Additional file 9 **Most reliable subset of fragments**. Maximum likelihood structures found by *MCMC5C *from the undifferentiated and differentiated THP-1 datasets (A and B, respectively). The HoxA gene transcription start sites are annotated on each of the structures. The most reliable fragment subset of size ten for each of the structures is indicated by shaded white circles. For both undifferentiated (fragments 2, 4, 19, 23, 30, 33, 37, 38, 40, and 41) and differentiated (fragments 2, 7, 15, 17, 21, 23, 24, 28, 33, and 38) states, the most reliable subset of fragments is concentrated at the center of the structure. Click here for file

Additional file 10 **Base Looping analysis of undifferentiated and differentiated THP-1 cells**. Analysis of base looping comparing the undifferentiated (red curve) and differentiated (blue curve) cell states. An ensemble of one hundred structures generated by *MCMC5C *was used for each state. The base looping measure was calculated with a sphere of radius one (1.0) every tenth base. The error bars report the standard deviation. Click here for file

Additional file 11 **Hi-C Human chromosome 14 movie**. A QuickTime movie of the Hi-C human chromosome 14 structure as determined by *MCMC5C *from previously published data [18]. Click here for file
