# Supplementary figures and images for: Three-dimensional modeling of chromatin structure from interaction frequency data using Markov chain Monte Carlo sampling
Source: BMC Bioinformatics. 2011 Oct 25;12:414. doi: 10.1186/1471-2105-12-414 (PMC3245522; doi:10.1186/1471-2105-12-414)

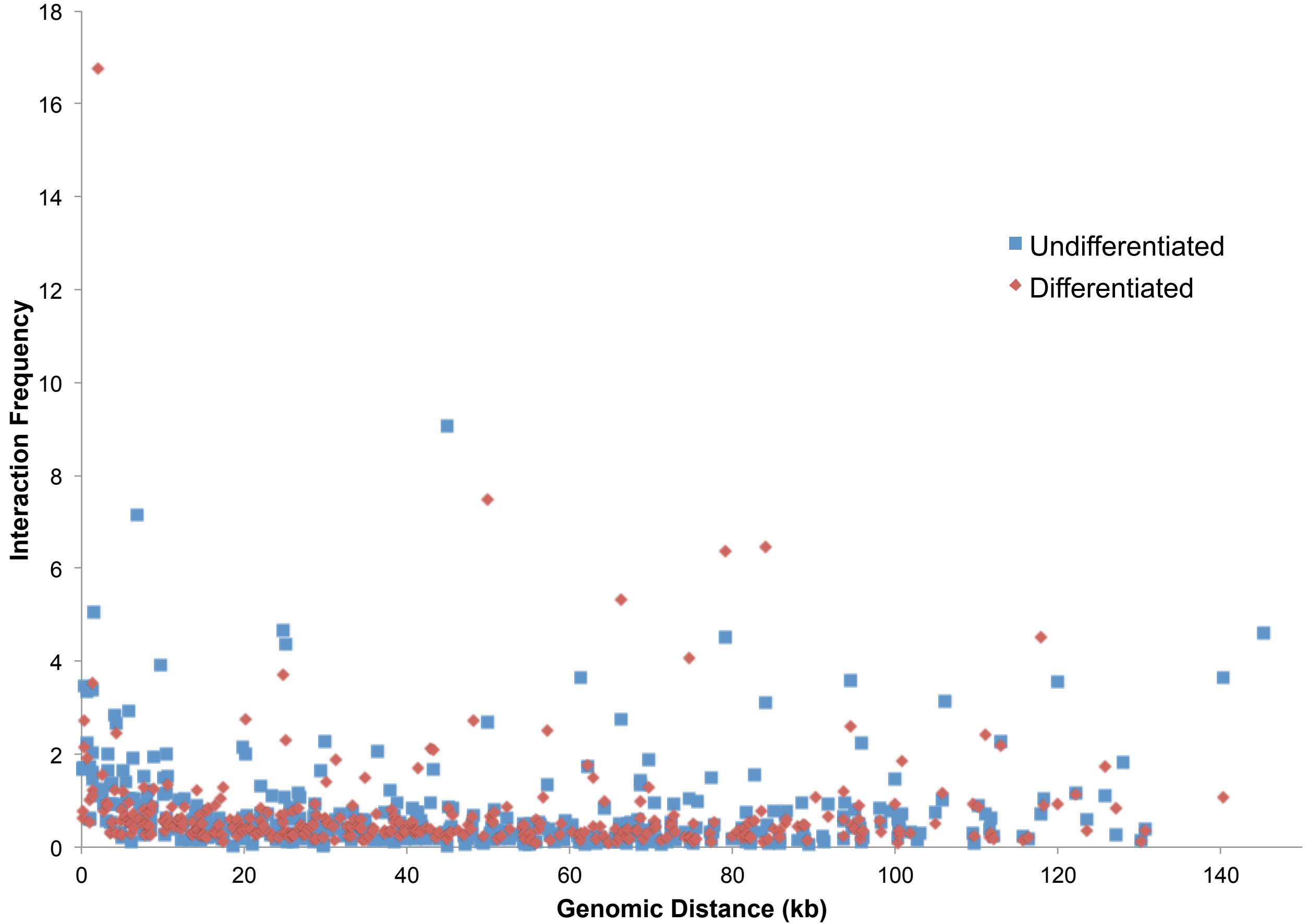

Supplement: Additional file 1 — Compaction profile of the HoxA region for THP-1 undifferentiated and differentiated cell states. Compaction profile of the HoxA cluster for both the undifferentiated (blue squares) and differentiated (red diamonds) THP-1 cell states. The average interaction frequency value diminishes with increasing linear genomic distance between the fragment pair, but strong contacts can be seen to exist between fragments at distances over 10-kb apart. [file 1471-2105-12-414-S1.PDF]

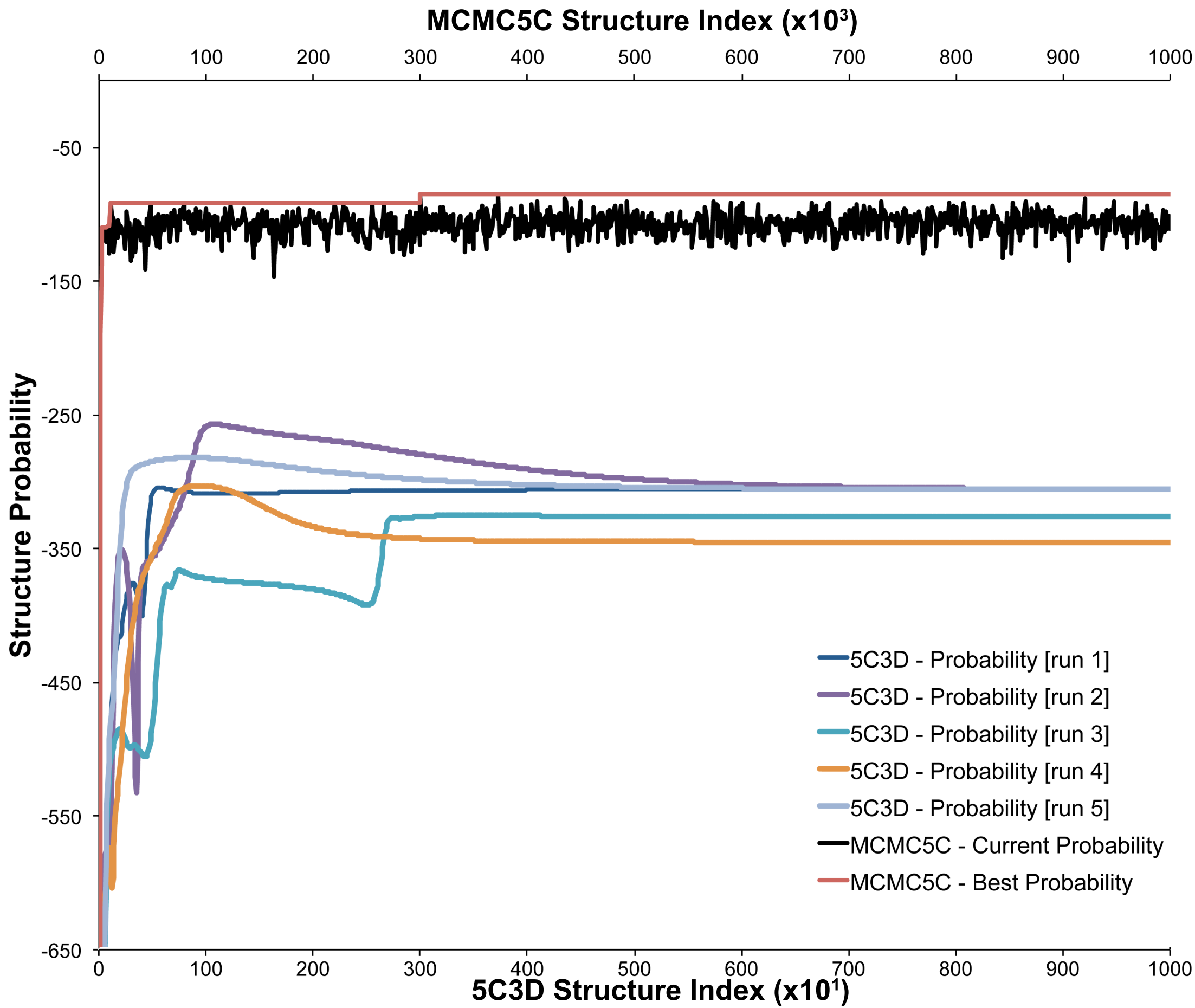

Supplement: Additional file 2 — HB-1119 Likelihoods of MCMC5C and 5C3D structures. Likelihood of the structures produced by MCMC5C and by several runs of 5C3D, as a function of the number of iterations (note the different scales of the x-axis for the two approaches). 5C3D very quickly converges to locally optimal structures that are slightly sub-optimal, and different runs converge to different solutions. [file 1471-2105-12-414-S2.PDF]

A

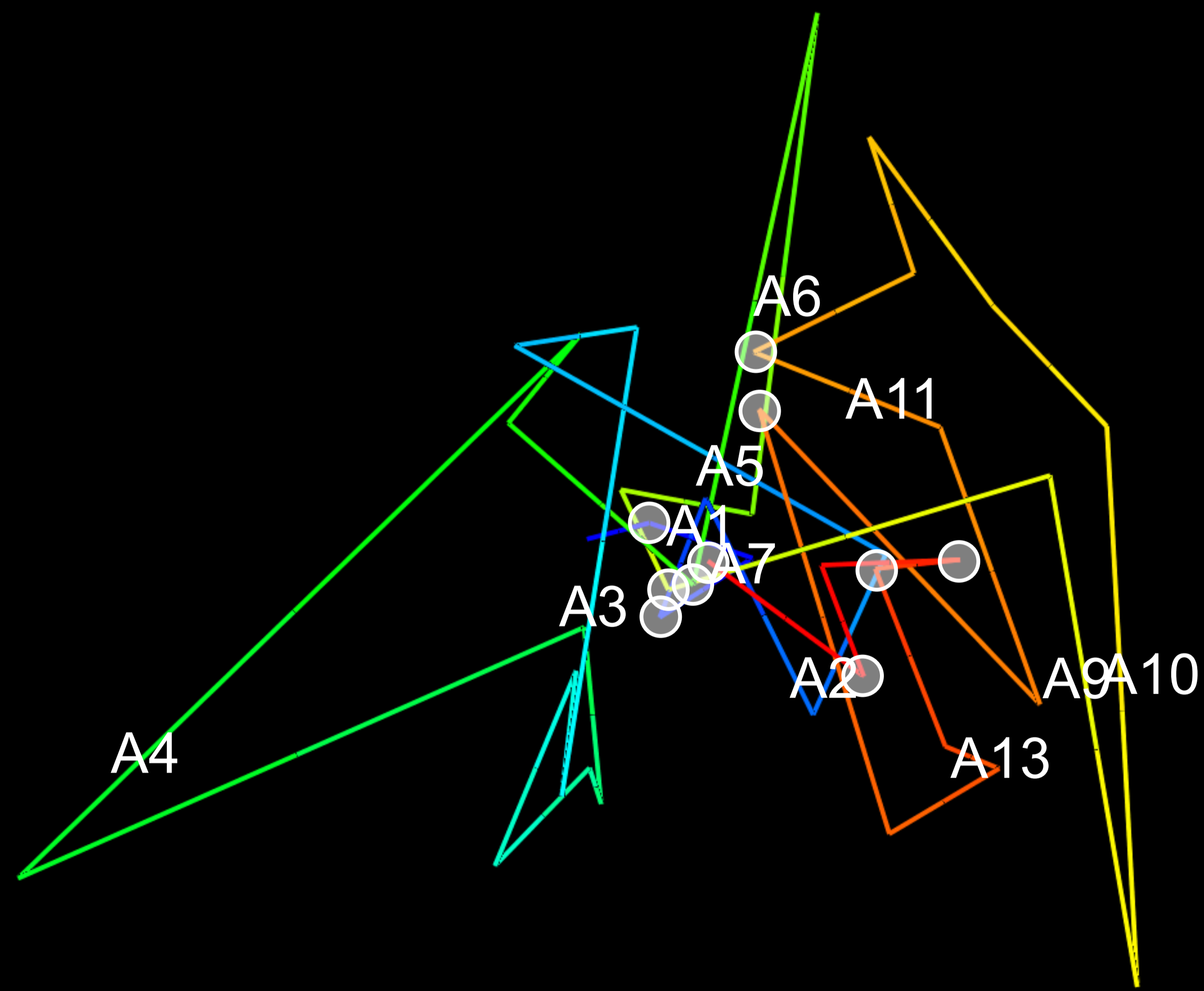

B

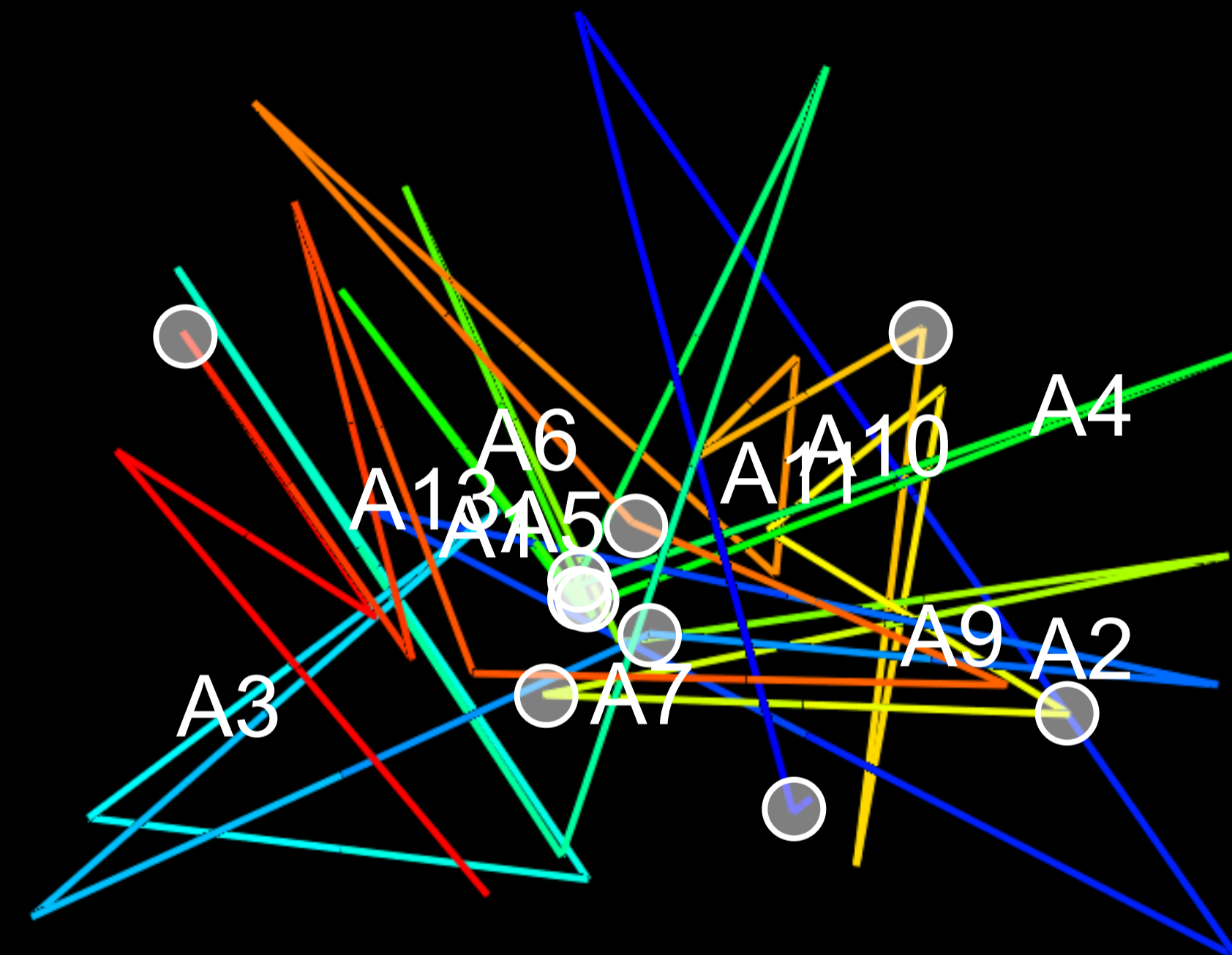

Supplement: Additional file 9 — Most reliable subset of fragments. Maximum likelihood structures found by MCMC5C from the undifferentiated and differentiated THP-1 datasets (A and B, respectively). The HoxA gene transcription start sites are annotated on each of the structures. The most reliable fragment subset of size ten for each of the structures is indicated by shaded white circles. For both undifferentiated (fragments 2, 4, 19, 23, 30, 33, 37, 38, 40, and 41) and differentiated (fragments 2, 7, 15, 17, 21, 23, 24, 28, 33, and 38) states, the most reliable subset of fragments is concentrated at the center of the structure. [file 1471-2105-12-414-S9.PDF]

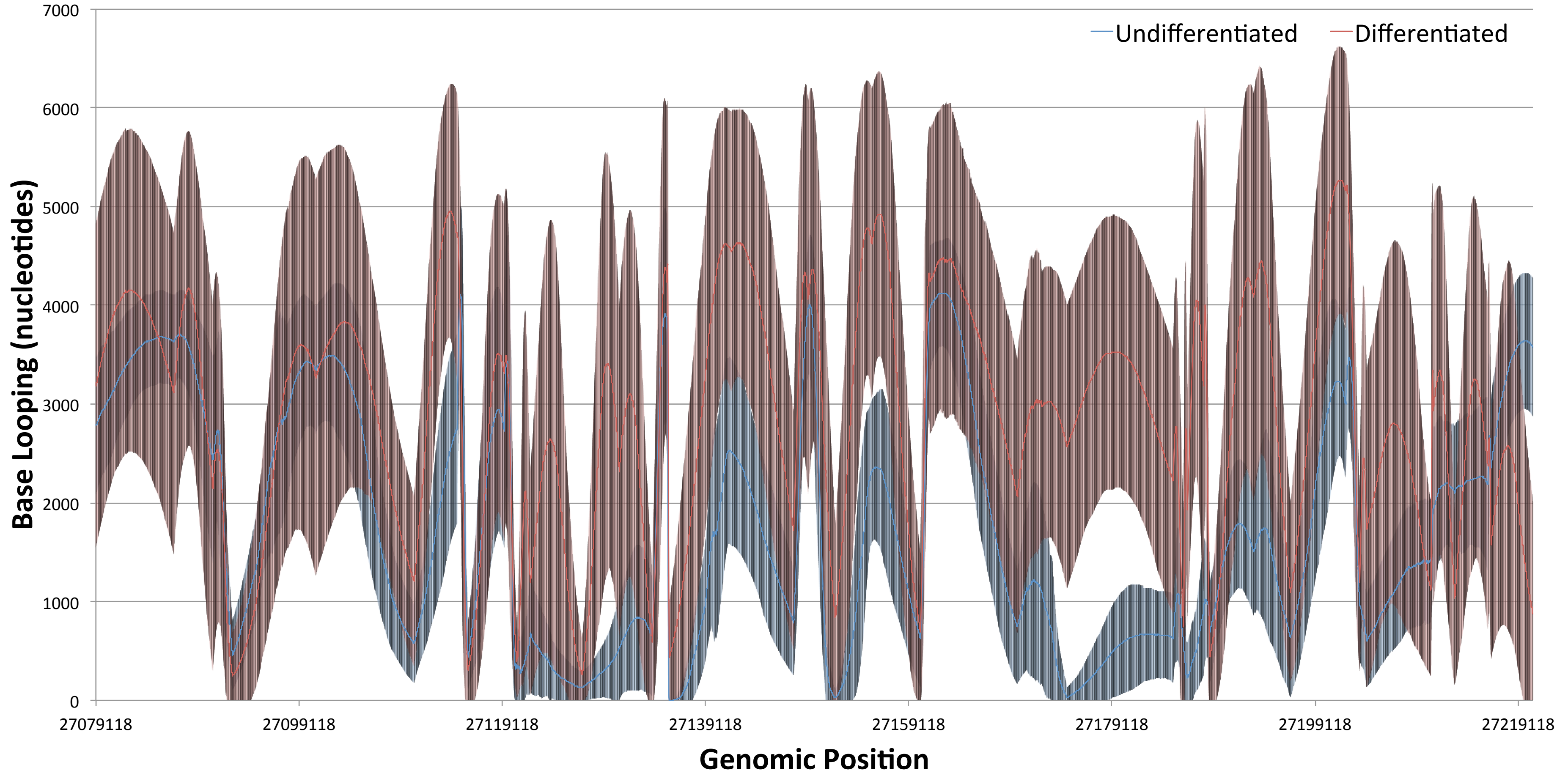

Supplement: Additional file 10 — Base Looping analysis of undifferentiated and differentiated THP-1 cells. Analysis of base looping comparing the undifferentiated (red curve) and differentiated (blue curve) cell states. An ensemble of one hundred structures generated by MCMC5C was used for each state. The base looping measure was calculated with a sphere of radius one (1.0) every tenth base. The error bars report the standard deviation. [file 1471-2105-12-414-S10.PDF]
